# Neuropathic midfoot deformity: associations with ankle and subtalar joint motion

**DOI:** 10.1186/1757-1146-6-11

**Published:** 2013-03-25

**Authors:** David R Sinacore, David J Gutekunst, Mary K Hastings, Michael J Strube, Kathryn L Bohnert, Fred W Prior, Jeffrey E Johnson

**Affiliations:** 1 Applied Kinesiology Laboratory, Program in Physical Therapy, Washington University School of Medicine, Campus Box 8502, Saint Louis, MO 63108, USA; 2 Department of Medicine, Washington University School of Medicine, St. Louis, MO, USA; 3 Motion Analysis Laboratory, Department of Orthopedic Surgery, Mayo Clinic, Rochester, MN, 55905, USA; 4 Department of Psychology, Washington University, Campus Box 1125, St. Louis, MO, USA; 5 Electronic Radiology Laboratory, Mallinckrodt Institute of Radiology, Washington University School of Medicine, St. Louis, MO, USA; 6 Department of Orthopaedic Surgery, Washington University School of Medicine, St. Louis, MO, USA

**Keywords:** Foot alignment, Deformity, Ankle and foot joint goniometry, Limited joint mobility

## Abstract

**Background:**

Neuropathic deformities impair foot and ankle joint mobility, often leading to abnormal stresses and impact forces. The purpose of our study was to determine differences in radiographic measures of hind foot alignment and ankle joint and subtalar joint motion in participants with and without neuropathic midfoot deformities and to determine the relationships between radiographic measures of hind foot alignment to ankle and subtalar joint motion in participants with and without neuropathic midfoot deformities.

**Methods:**

Sixty participants were studied in three groups. Forty participants had diabetes mellitus (DM) and peripheral neuropathy (PN) with 20 participants having neuropathic midfoot deformity due to Charcot neuroarthropathy (CN), while 20 participants did not have deformity. Participants with diabetes and neuropathy with and without deformity were compared to 20 young control participants without DM, PN or deformity. Talar declination and calcaneal inclination angles were assessed on lateral view weight bearing radiograph. Ankle dorsiflexion, plantar flexion and subtalar inversion and eversion were assessed by goniometry.

**Results:**

Talar declination angle averaged 34±9, 26±4 and 23±3 degrees in participants with deformity, without deformity and young control participants, respectively (*p*< 0.010). Calcaneal inclination angle averaged 11±10, 18±9 and 21±4 degrees, respectively (*p*< 0.010). Ankle plantar flexion motion averaged 23±11, 38±10 and 47±7 degrees (*p*<0.010). The association between talar declination and calcaneal inclination angles with ankle plantar flexion range of motion is strongest in participants with neuropathic midfoot deformity. Participants with talonavicular and calcaneocuboid dislocations result in the most severe restrictions in ankle joint plantar flexion and subtalar joint inversion motions.

**Conclusions:**

An increasing talar declination angle and decreasing calcaneal inclination angle is associated with decreases in ankle joint plantar flexion motion in individuals with neuropathic midfoot deformity due to CN that may contribute to excessive stresses and ultimately plantar ulceration of the midfoot.

## Background

Neuropathic midfoot deformities impair foot and ankle joint mobility, often leading to abnormal stresses and impact forces during walking [1]. The sequelae of impaired foot or ankle joint motion coupled with excessive plantar stresses in individuals with diabetes mellitus (DM) and peripheral neuropathy (PN) are ulceration, infection and ultimately lower extremity amputation [2]. Neuropathic midfoot deformities present a formidable challenge to orthopaedic, podiatric and rehabilitation specialists, since these deformities are multi-planar, insidious in onset, and most difficult to attribute and recognize an incipient cause. Neuropathic midfoot deformities are unremitting in their progression, and thus present an ever-increasing risk for foot amputation [3].

Charcot neuroarthropathy (CN) has often been believed to be a precipitating event preceding mid tarsus deformities [3,4] though the direct causal link remains elusive since protean bone and joint degeneration progresses over a seemingly variable time course [3].

Neuropathic midfoot deformities have been commonly demonstrated by radiographic measures that exceed bone and joint alignment values for individuals without deformities [5-7]. Schon and colleagues have reported that neuropathic individuals with acquired mid tarsus deformities and accompanying plantar ulcerations occur when hind foot alignment exceed the values encountered in asymptomatic, non-neuropathic adults without midfoot deformities [5]. Bevan and Tomlinson have shown that excessive foot radiographic alignment measures are found in diabetic individuals with midfoot ulceration compared to diabetic individuals without midfoot ulceration [8].

Limited joint mobility (LJM) in the foot and ankle has previously been linked to neuropathic ulceration [9-11] and excessive plantar pressures [10,11]. Limited ankle joint and subtalar joint mobility can impair foot function and contribute to excessive vertical plantar pressures leading to neuropathic ulceration [1]. However, there has been no previous report of the association of mal-alignment of the tarsal bones to ankle and subtalar joint mobility in neuropathic participants with or without plantar ulcerations. The impact of LJM in the ankle and subtalar joints to the onset and progression of acquired mid tarsus deformities is unknown.

Based on our clinical observations and previous studies of patients with CN [12,13], we suspected that ankle joint plantar flexion motion may be impaired in participants with neuropathic midfoot deformities but could find no previous reports confirming our clinical observations. Therefore, the purposes of this study were to: (i), assess radiographic measures of hind foot alignment and ankle joint (AJ) and subtalar joint (STJ) motion in participants with and without neuropathic midfoot deformities due to CN; and (ii), determine the relationship between radiographic measures of hind foot alignment and AJ and STJ range of motion. We tested the null hypothesis that there would be no differences in AJ or STJ motion in participants with diabetes and peripheral neuropathy with and without neuropathic midfoot deformities compared to young control participants. Similarly, we hypothesized there would be no differences in the relationships between AJ or STJ motion and radiographic measures of hind foot alignment in participants with or without neuropathic midfoot deformity.

## Methods

Sixty participants were studied in three groups. Participants were selected for study based on the inclusion and exclusion criteria previously described [14]. Forty participants had DM and PN (19 men, 21 women, mean age = 57 ± 10 yrs). Twenty of these participants (n=20) with DM and PN had a unilateral CN with midfoot deformity, while twenty (n=20) participants with DM and PN had neither CN or midfoot deformity These two groups of participants with DM and PN were compared to a group of 20 younger, control participants (11 men, 9 women, mean age = 28 ± 6 yrs) without DM, PN, CN or foot deformity.

The participants’ CN was diagnosed by their physician (e.g. either their primary care physician or community orthopedist) based on history, clinical examination of signs and symptoms of an acute inflammation and standard weight bearing foot radiographs at the time of their first presentation [3]. The CN was temporally staged using the Eichenholtz classification system [15] with the addition of stage 0 proposed by Shibata et al. [16]. Stage 0 is the acute clinical phase in which the foot is warm, swollen, and red but there is limited or no radiological evidence of fracture. Stage I is the fragmentation/dislocation phase in which the clinical signs continue but fractures, joint dislocations, instability, and deformity become readily visible on standard radiological examinations. Stage II is the coalescence phase in which there is resorption of bone fragments and fusion of larger bone fragments. Stage III is the consolidation phase in which the foot becomes stable [15]. All participants had assessments to determine the presence of neuropathy, ankle joint and subtalar joint goniometry and weight-bearing foot radiographs. Participants with DM and PN had their blood drawn to determine the percent of glycated hemoglobin. Participants with neuropathic midfoot deformity had all assessments performed at their initial visit prior to any intervention or management of CN.

### Neuropathy and glycated hemoglobin

All participants had testing at nine locations on the plantar surface of each foot using 3 thicknesses of Semmes Weinstein monofilaments, the 4.17 (1-gram), 5.07 (10-gram) and 6.10 (75-gram) monofilaments [17,18]. If the participant was able to accurately sense the 4.17 monofilament at all nine locations, sensation was graded as normal. If the participant was able to sense the 6.10 monofilament in at least one plantar location but unable to feel the 5.07 filament in at least one location, the sensation was graded as diminished, and if unable to sense the 6.10 filament at any single location, the sensation was graded as absent. Any grade of diminished or absent sensation confirmed the loss of protective sensation and the participant was judged to have peripheral neuropathy.

Vibration perception threshold (VPT expressed as volts) was assessed in all participants with DM to confirm the presence or absence of vibration sensation in their feet. We used a Bio-thesiometer (Bio-Medical Instrument Co, Newbury OH) with the probe held perpendicular to the plantar surface of the distal great toe. In general, a VPT score greater than 25 V is consistent with impaired vibration sense and the presence of peripheral neuropathy [17].

All participants with DM and PN had serum percent of glycated hemoglobin (HbA1c) assessed confirming their diagnosis of diabetes mellitus. Since young control participants did not have DM, PN or foot deformity, control participants did not have their HbA1c or their vibration perception threshold assessed and assumed to have normal age-appropriate values.

### Ankle and subtalar joint goniometry

Ankle joint dorsi flexion (AJ DF) and plantar flexion (AJ PF) and subtalar joint inversion (INV) and eversion (EVR) were assessed using a standard plastic full-circle goniometer (2 degree intervals) with each subject in the prone position with the foot and ankle overhanging the end of the table. For active AJ DF and AJ PF, the axis of the goniometer was placed just distal to the lateral malleolus; the stationary arm was aligned with the bisection of the lateral leg and moveable arm aligned with the plantar surface of the heel pad. The plane of the plantar surface of the foot and the 5th metatarsal were not used as landmarks to align the moveable arm when assessing AJ DF motion since excessive midfoot motion would overestimate the amount of true AJ motion. For STJ INV and EVR, the axis was placed immediately above the posterior calcaneus, the stationary arm was aligned with the bisection of the posterior leg and the moveable arm aligned with the bisection of the posterior surface of the calcaneus [18]. The same experienced clinician made all goniometric measurements. It is acknowledged that AJ and STJ are tri-planar joints meaning each allows simultaneous movement in all 3 cardinal planes of motion, though clinical goniometry assesses the amount of active and passive movement in one single plane that is often representative of the largest motion. The reliability and intra-rater agreement for select goniometric motions has been previously established [18-20] with reliability coefficients for intra-rater determinations ranging from 0.89 to 0.96 for AJ DF and STJ INV and EVR with standard error of the measurements ranging from 1 degree (STJ eversion) to 3 degrees (STJ inversion) [19]. Intra rater reliability of AJ PF reliability has been reported to range from 0.47 to 0.99 in published reports [20].

### Radiographic alignment

**Figure 1:**
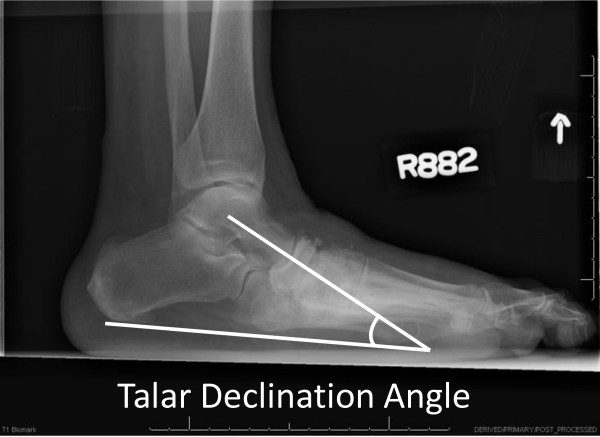
Talar declination angle formed by the line representing the collum tali axis through the head and neck of the talus and a line representing the weight bearing plantar surface from the calcaneus to the 5th metatarsal.

**Figure 2:**
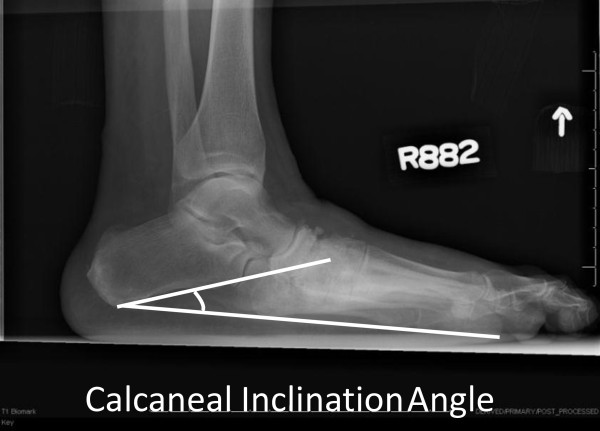
Calcaneal inclination angle formed by the line representing the calcaneal inclination axis drawn from the anterior, inferior calcaneal border of the calcaneocuboid articulation and a line representing the weight bearing plantar surface from the calcaneus to the 5th metatarsal.

All participants had standardized weight-bearing radiographs of both feet (antero-posterior, lateral and oblique views). A research assistant monitored and standardized each foot position for image consistency and to minimize out-of-plane rotations [3]. A calibration ruler was included in each radiograph to calibrate each image and reduce geometric magnification errors. Radiographs were de-identified of all personal information then uploaded and imported into ISite PACS (Picture Archiving and Communication Systems) workstation software program for measurement (Philips Healthcare Informatics, Foster City, CA). The ISite PACS software measures angles to the nearest 1-degree increment. Talar declination and calcaneal inclination angles were assessed on the lateral view using the angle measurement function in the ISite PACS software. Standard view foot radiographs demonstrate single plane foot deformities. Talar declination and calcaneal inclination angles were selected to represent hind foot alignment changes in the sagittal plane that are best represented by the lateral view foot radiograph. The talar declination angle was measured as the angle formed between the collum tali axis, a line originating from the center of the body of the talus extended through the bisection of the talar neck and head with a horizontal line extending from the plantar surface of the calcaneus to the plantar surface of the 5th metatarsal head [21] (Figure 1). Talar declination angle was measured since it reflects the orientation of the distal head and neck of talus in the sagittal plane and is impaired in flat feet deformities [5]. Calcaneal inclination (pitch) angle was measured as the angle formed between lines extending from inferior portion of the calcaneocuboid joint to the same horizontal line along the plantar aspect of the calcaneus to the plantar surface of the 5th metatarsal head (Figure 2). Calcaneal inclination angle was measured since it reflects the orientation of the anterior calcaneus in the sagittal plane and is impaired in flat feet. The same rater performed radiographic measurements. As reported previously, measurement error as the standard deviation between repeat measurements is 2 degrees for both talar declination angle and calcaneal inclination angles in women 40 to 60 years of age [22]. Hastings et al. recently reported the intra-rater measurement precision of radiographic measures expressed as the root mean square standard deviation (RMS-SD) in participants with DM and PN and foot deformity is 2 to 3 degrees [23]. Both angle measures have been used to describe the alignment of the hind foot bones in the sagittal plane and reflect mid tarsal joint (talonavicular and calcaneocuboid) alignment in acquired neuropathic midfoot deformities [3,5].

Qualitative assessments of foot radiographs by an expert clinician (JEJ) were used to identify participants with observable mal-alignments (e.g. partial subluxation or complete dislocation) of either the talonavicular (TN) joint or calcaneocuboid (CC) joint. A complete dislocation was operationally defined as ≥ 50% joint surface incongruence with bones mal-aligned, whereas a partial subluxation was operationally defined as < 50% of joint surface incongruence of the mal-aligned bones on any view of the foot radiograph. The mean value of AJ and STJ motion in this small subset of participants with DM, PN and observable mal-alignments are reported separately and contrasted to the mean values of the DM PN participants without deformity in order to relate the goniometric impairment to the radiographic alignment.

### CN management

Medical management of CN was determined by each participant’s care provider. Immobilization and offloading was through total contact casting transitioning to a removable-cast walker boot for ten participants and a removable-cast walker boot exclusively for six participants [3]. Four participants alternated between total contact casting and a removable-cast walker boot throughout the duration of offloading. Nine CN subjects received treatment from one of the authors (DRS), and the remaining subjects received treatment from providers not associated with the study (e.g. primary care physicians, community orthopedists). The length of time for immobilization and offloading was variable as determined by the primary care physicians or community orthopedists. The average length of time of immobilization for all participants was 19 ± 10 weeks [3].

All participants read and signed an informed consent that outlined the research protocol risks and their agreement to participate. Our research protocol (IRB ID# 201103036) was approved by our institutional review board at Washington University School of Medicine St Louis MO. Participants were remunerated for their participation.

### Data analysis

Group means, standard deviations, upper and lower 99% confidence limits around the mean values were calculated for radiographic and goniometric measures. Group demographic characteristics were compared using Chi square test for sex, diabetes type, race and presence of neuropathy with S-W monofilaments. A one-way analysis of variance (ANOVA) was used to compare the groups’ age, height, weight, body mass index, HbA1C, duration of diabetes mellitus and vibration perception threshold. A one-way ANOVA was used to determine the difference in mean values for goniometric and radiographic measures. In these analyses, both feet were averaged for the Young Control and DM PN No Deformity groups and compared to the involved foot for the DM PN with deformity group. Post-hoc pair-wise contrasts were conducted using t-tests. A Bonferroni correction was applied to reduce the alpha level and protect against a Type 1 error for the number of post-hoc comparisons. A paired t-test was used to compare mean values between feet within each group (right versus left feet in the Young Control and DM PN No Deformity groups; involved versus uninvolved feet in the DM PN with deformity group). Pearson correlation coefficients were used to determine the association between radiographic alignment and goniometric measures with all participants pooled and within individual groups with participants’ feet combined. The pooled correlations were residualized to remove the influence of group mean differences and so represent a purer estimate of the relationship between variables across all participants. All statistical analyses were completed using Statistical Package for the Social Sciences (SPSS) version 19 (IBM, Chicago, IL).

## Results

Control participants were younger and weighed less than participants with DM and PN with and without deformity. Participants with CN deformity were heavier with greater BMI than DM PN participants without CN deformity but did not differ in other physical characteristics or disease-related characteristics studied. In 20 participants with DM PN and CN midfoot deformity, 18 participants were classified as Eichenholtz stage I and two participants were classified as stage II. Using the Schon anatomical location classification system [5], seven participants had involvement at the Lisfranc joints (Type 1) and five participants had involvement at the transverse tarsal joints (Type 4). Two participants had naviculo-cuneiform (Type II) involvement, one had peri-navicular involvement (Type III), and 5 participants had mixed (multiple) joint involvement. Nine participants had deformity in the right foot and 11 had deformity in the left foot (Table 1). Four participants (20%) had a concurrent plantar ulceration on the same side as the midfoot deformity, 2 participants (10%) had concurrent plantar ulceration on the contra-lateral side. Five participants (25%) had previously reported a plantar ulceration in the 3 years preceding this study in the foot with midfoot deformity. No participant in the DM PN without deformity group had an active plantar ulceration, and none had a history of osteomyelitis or midfoot ulceration. There were no severe digit or forefoot deformities, though several participants had mild claw and hammer toe deformities consistent with intrinsic muscle weakness.

**Table 1:** Group demographics and physical characteristics

**Characteristic**	**DM PN and deformity**	**DM PN No deformity**	**Young controls**	**p value**
Age (years)	55 ± 9	58 ± 11	28 ± 6	<0.05
Race	4 Black	4 Black	2 Black	NS
	16 Caucasian	15 Caucasian	16 Caucasian	
		1 Asian	2 Asian	
Gender (M/F)	10 / 10	9 / 11	11 / 9	NS
HT (cm)	175 ± 8	172 ± 8	173 ± 10	NS
WT (kg)	113 ± 26	95 ± 26	79 ± 18	<0.05
BMI (kg/m^2^)	37 ± 7	32 ± 8	26 ± 5	<0.05
DM Type (1/2)	2 / 14	3 / 16	N/A	NS
DM duration (years)	16 ± 10	15 ± 13	N/A	NS
Touch/pressure to S-W monofilament (No.)	Absent	Diminished (4)	Normal (20)	NS
	(20)	Absent (16)		
VPT (volts)	33 ± 12	27 ± 12	N/A	NS
HbA1c (%)	7.8 ± 1.8	7.8 ± 1.4	N/A	NS
Eichenholtz stage (No. I/II)	18/2	N/A	N/A	
Schon classification (No. Type I/II/III/IV/mixed)	7/2/1/5/5	N/A	N/A	
Deformity foot (R/L)	9/11	N/A	N/A	

S-W monofilament is Semmes-Weinstein monofilament; VPT is vibration perception threshold; HbA1c is percent of glycated hemoglobin. Eichenholtz Stage is temporal staging by clinical and radiographic criteria. Schon Classification is location of deformity; Type I is Lisfranc joints; Type II is naviculo-cuneiform; Type III is peri-navicular and Type IV is transverse tarsal joints. NS is not significant. N/A is not applicable.

Talar declination angle was increased in the participants with midfoot deformity compared to both other groups (p<0.05). Calcaneal inclination angle was decreased in participants with midfoot deformity compared to both other groups, but significantly different only relative to the young control group. There were no differences in hind foot alignment angles between left and right feet of participants with DM PN without deformity or young control participants.

AJ DF motion was greatest in young control participants compared to both groups of participants with DM and PN. There was no difference in AJ DF motion in groups of participants with DM, PN with or without CN deformity. The mean AJ PF motion was lowest on the DM PN foot with deformity compared to DM PN participants without CN deformity and young control participants’ feet (*p* < 0.001). The mean AJ PF in participants with DM PN without CN deformity was significantly lower than the mean values for young control participants. The involved foot of participants with DM PN with CN deformity had an increased talar declination angle, and limited motion in AJ PF and STJ inversion compared to the uninvolved, contra-lateral foot (Table 2).

**Table 2:** Mean ± SD and 99% lower and upper confidence limits of goniometric and radiographic measures

	**DM, PN and deformity**		**DM PN No deformity**	**Young control**	**Group *p *value**
	**Involved**	**Uninvolved**			
AJ Plantar-flexion (degree)	23 ± 11*	35 ± 15	38 ± 10	47 ± 7	< .001
	15 - 30	25 - 45	32 - 45	43 - 52	
AJ Dorsi-flexion (degree)	5 ± 6	7 ± 6	7 ± 5	12 ± 3	< .001
	1 - 9	3 - 10	3 - 10	9 - 14	
STJ inversion (degree)	11 ± 6*	15 ± 7	15 ± 5	16 ± 3	< .001
	8 - 15	11 - 20	12 - 19	13 - 18	
STJ eversion (degree)	5 ± 2	5 ± 3	7 ± 2	8 ± 1	< .001
	4 - 7	3 - 8	6 - 9	7 - 9	
Talar declination angle (degree)	34 ± 9*	30 ± 5	26 ± 4	23 ± 3	< .003
	28 - 40	27 - 33	23 - 28	22 - 25	
Calcaneal inclination angle (degree)	11 ± 10*	16 ± 8	18 ± 9	21 ± 4	< .001
	5 - 18	11 - 22	13 - 24	18 - 24	

Values denoted with * are different than their paired foot, (*p*<0.05). DM, PN and Deformity is diabetes mellitus, peripheral neuropathy and deformity; AJ is ankle joint; STJ is subtalar joint. Group p values refers to the comparisons of the three participant groups using the average of both feet for Young Control and DM PN No Deformity groups and the Involved Foot for the DM PN Deformity group. Comparisons to the Uninvolved foot for the DM PN Deformity group were significant for AJ Plantar-flexion, AJ Dorsi-flexion, STJ Eversion and Talar Declination Angle (*p* <0.01).

**Figure 3:**
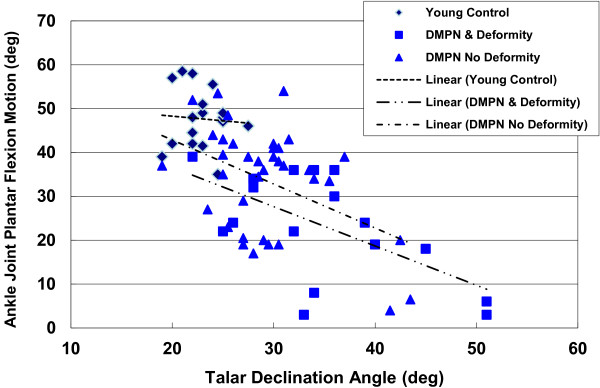
Relationship between talar declination angle and ankle joint plantar flexion motion by group of participants.

**Figure 4:**
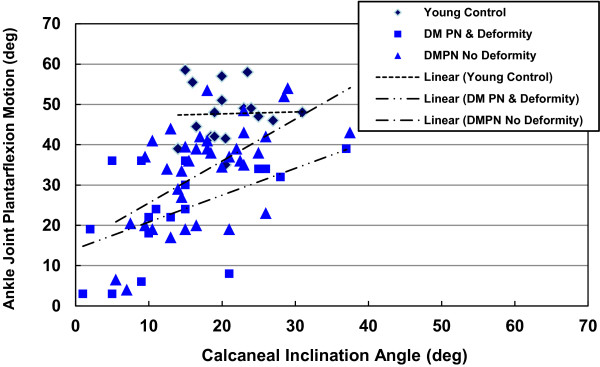
Relationship between calcaneal inclination angle and ankle joint plantar flexion motion by group of participants.

We found an inverse relationship between loss of AJ plantar flexion motion and increasing talar declination angle (Table 3, Figure 3) and a direct relationship between loss of AJ plantar flexion motion and decreasing calcaneal inclination angle (Table 3, Figure 4) in participants (group data pooled). However, these relationships are most influenced by the involved foot in the participants with DM, PN, and foot midfoot deformity (r = −0.476 for talar declination angle, r = 0.541 for calcaneal inclination angle; Figures 3 and 4). We did not observe as strong relationships between talar declination or calcaneal inclination angles with AJ dorsiflexion or STJ inversion and eversion motions in any groups or feet examined (Table 3). The relationships between talar declination angle, calcaneal inclination and AJ PF motions in all groups of participants are shown in Figures 3 and 4, respectively.

**Table 3:** Correlation coefficients of goniometric and radiographic alignment measures

**Group**	**Variable**	**AJ**	**AJ**	**STJ**	**STJ**
		**DF**	**PF**	**INVER**	**EVERS**
		**ROM**	**ROM**	**ROM**	**ROM**
All groups	Talar Dec	0.210	−0.348	−0.063	0.122
	Calcan Inc	−0.028	0.397	−0.133	0.059
Young control	Talar Dec	−0.303	−0.230	−0.057	0.136
	Calcan Inc	−0.213	−0.100	−0.229	−0.062
DM PN	Talar Dec	0.163	−0.155	0.128	0.024
No deformity	Calcan Inc	0.166	0.388	−0.324	−0.170
DM PN and deformity involved foot	Talar Dec	0.324	−0.476*	−0.163	0.155
	Calcan Inc	−0.127	0.541*	0.062	0.277

Pearson correlations coefficients (r) between radiographic alignment and goniometric measures within groups with left and right feet averaged for Young Control and DM PN and No Deformity groups. Correlations are residualized for all groups combined to eliminate bias due to mean differences. * *p* < 0.05.

Participants with a complete dislocation of either the TN or CC joints have a profound restriction in AJ PF motion (greater than 50% restriction) and to a lesser extent STJ inversion and eversion motions (40% restriction) contrasted to participants without these joint dislocations (Table 4).

**Table 4:** Mean ± SD of radiographic and goniometric measures for participants with talonavicular (TN) and calcaneocuboid (CC) dislocation or subluxation

	**TN**	**CC**	**TN**	**CC**
	**Dislocation**	**Dislocation**	**Subluxation**	**Subluxation**
No. of participants/No. of feet	7 / 9	4 / 4	4/ 5	11/ 12
AJ Plantar flexion (deg)	14 ± 9	13 ± 9	38 ± 7	32 ± 15
AJ Dorsi-flexion (deg)	7 ± 4	7 ± 3	8 ± 3	6 ± 3
STJ Inversion (deg)	9 ± 5	9 ± 7	16 ± 5	16 ± 5
STJ Eversion (deg)	5 ± 3	5 ± 3	8 ± 2	8 ± 3
Talar Declination Angle (deg)	40 ± 8	42 ± 11	30 ± 5	31 ± 7
Calcaneal Inclination Angle (deg)	7 ± 10	0 ± 10	21 ± 8	14 ± 4

## Discussion

The major findings of this study are: (i) both radiographic hind foot alignment measures and goniometric measures of AJ plantar flexion and STJ inversion are markedly different in participants with DM, PN and CN midfoot deformity; and (ii) both radiographic hind foot measures of alignment are associated with restriction of AJ PF range of motion, particularly in the involved foot of participants with DM PN and CN midfoot deformity.

Talar declination angle is typically 21 to 24 degrees [21]. In feet with CN midfoot deformity, talar declination angle was markedly greater than either the contra-lateral feet without CN deformity or feet of participants with DM and PN and no deformity and the young control group. It has been reported that collum tali angles declining to greater than 29 degrees are excessive and more frequently associated with mid tarsal joint destruction, non-reducible pronation deformities and sequelae including plantar ulceration along the medial or central columns of the foot [5,8].

Calcaneal inclination (pitch) angle is typically 18 to 22 degree [21]. A calcaneal inclination angle less than 18 degrees is more frequently associated with mid tarsal joint destruction, pronation deformities and sequelae including plantar ulceration [8]. The impact of markedly greater talar declination angles and lower calcaneal inclination angles in participants with midfoot deformity during walking is not known and await further study.

Our finding of LJM of AJ DF was not surprising. As reported previously [19,24-26], we have found limited AJ DF motion in all participants with DM and PN but find no major differences in restriction of AJ DF motion between participants with DM and PN with or without midfoot deformity. However, the profound limitation in AJ PF motion and a lesser restriction in STJ inversion motion in the involved foot of participants with DM, PN and CN midfoot deformity is novel. Participants with DM PN and midfoot deformity lost more than 50% of AJ plantar flexion motion and 30% of STJ inversion motion compared to young control participants. The loss of AJ plantar flexion and STJ inversion may impair foot function during walking and contribute to excessive stress and impact loading that may lead to structural collapse of the midfoot and hind foot [1,27]. Neuropathic individuals with major restrictions in AJ and STJ motion may be (unknowingly) forced to alter their gait patterns. Limited AJ PF motion reduces their ability to push off during walking. Along with limited AJ DF, they may compensate by increasing the foot progression angle and excessively loading the medial column of the midfoot [28]. We have observed an abnormal transfer of stress and impact loads to the plantar surface of the midfoot in individuals with neuropathic deformities that readily contributes to midfoot ulceration [1]. Neuropathic individuals with restrictions in AJ and STJ motion should be examined thoroughly and monitored frequently by their physicians and rehabilitation specialists to assess gait patterns that may contribute to plantar ulceration or further midfoot collapse that may ultimately lead to amputation.

The underlying incipient cause of the loss of ankle joint plantar flexion and subtalar joint inversion motion in individuals with neuropathic foot deformities and hind foot mal-alignments is not known. It is likely that once the talus declines, the tibiotalar joint surfaces become more impinged with a loss of joint space that restricts plantar flexion. As midfoot deformities increase, eventually the talus cannot plantar flex further because the posterior aspect of the talus has impinged against the tibia. Any observed foot plantar flexion motion may not be true ankle motion but rather a compensatory motion at other joints (e.g., the talonavicular or mid tarsal joints). A small number of participants’ feet (4/20, 20%) had excessive stress-induced osteophyte build up at the posterior tibia and talus that were visible on the radiograph that may also contribute to limit AJ plantar flexion motion.

An alternative explanation for the AJ plantar flexion restriction is soft tissue changes as a result of acute CN in adjacent joints and tissues, stiffness with reduced elasticity and excessive shortening of the Achilles tendon, combined with insufficiency of the plantar fascia, plantar ligaments (e.g. spring ligament) and posterior tibialis muscle and tendon as key contributors. These structures have been implicated in adult-acquired flat foot deformities [6,27] though the resultant foot deformities are not rigidly fixed and the ankle joint and subtalar joint motions do not appear to be as severely restricted. The incipient contributors of AJ and STJ motion restrictions require additional study to identify the precipitating events, the time course of onset as well as the most effective treatments to improve foot function and reduce plantar stresses.

The utility of relating impairments in joint motions to weight bearing structural alignment may be early detection of individuals with high-risk neuropathic feet. We studied a small number of participants with complete or partial talonavicular (TN) and calcaneocuboid (CC) joint dislocations. Those participants with complete TN dislocation had an excessive talar declination angle (mean = 40±8 degrees) and low calcaneal inclination angle (mean = 7±10 degrees; Table 4) and severely restricted AJ plantar flexion and STJ inversion motion. Similarly, participants with complete CC joint dislocation had an abnormally excessive talar declination angle (mean = 42±11 degrees) with low calcaneal inclination angle (mean = 0 ±10 degrees) with severely impaired AJ plantar flexion and STJ inversion motion. While our data are limited by small numbers of participants, these observations may provide a focus for future investigations of individuals with LJM at high risk for midfoot ulceration and eventual amputation. In our sample of 20 participants with severely restricted AJ and STJ motion in the presence of foot neuropathic deformity, there were a high percentage of feet (9/20, 45%) with current or previously reported midfoot plantar ulcers due to excessive plantar pressures in the presence of midfoot deformities. These neuropathic participants with severely limited foot motion may be at the highest risk for plantar ulceration. Moreover, talar declination angles that exceed 30 degrees are more often indicative of partial or complete talonavicular dislocations with resulting medial column deformities; while calcaneal inclination angles less than 10 degrees are more often indicative of calcaneocuboid mal-alignments and lateral column deformities (Table 4). Individuals with DM and PN with radiographic talar declination and calcaneal inclination angles that exceed these values may be more apt to develop midfoot ulceration due to progression of plantar bony prominences [3,5,8].

There are limitations to our study. The young controls were nearly 30 years younger than participants with DM and PN. We chose to study a younger group of control participants to determine radiographic and goniometric values in those without neuropathy, foot deformity and diabetes or motion restriction. We speculate that participants with DM, PN and CN may have had goniometric AJ and STJ values and radiographic hind foot values similar to the control participants prior to onset of DM, PN and CN. Our static radiographic hind foot measures only describe the primary sagittal plane orientation of the talus and calcaneus after care is taken to align the foot during radiographic examination. We did not attempt to describe the frontal and transverse plane orientations of these hind foot bones using radiographs or other imaging modalities. Since many neuropathic deformities are multi-planar, they are challenging to identify and measure with precision and accuracy from standard weight bearing radiographs even for the most experienced foot and ankle clinician [3,23]. Other ankle and hind foot alignment views may better inform of ankle and foot function (e.g. STJ motion) though all physicians do not routinely obtain these views. The recognition of multi-plane neuropathic deformities though challenging, may be improved with other imaging tools such as computed tomography and magnetic resonance imaging that allow deformities to be characterized in three dimensions.

A last limitation is we were unable to assess mid tarsal joint motion directly with clinical goniometry due to imprecision and poor reproducibility. Clinical goniometry of the foot and ankle joints is most accurate for describing a single plane motion during complex tri-planar motions at the ankle and subtalar joints. Though impaired mid tarsal motions are likely impacted by motion restrictions at the ankle joint and the subtalar joint [29] and no doubt contribute to progressive mid tarsus joint deformities [3], mid tarsal motion cannot be assessed with the current methods.

## Conclusions

Neuropathic midfoot deformities as indicated by excessive talar declination angle and calcaneal inclination angle are associated with limited AJ plantar flexion and STJ inversion motion. As talar declination angle increases and calcaneal inclination decreases, ankle joint plantar flexion motion decreases in individuals with neuropathic midfoot deformities. The most severe restrictions in motion are observed in participants with complete talonavicular or calcaneocuboid dislocations. Goniometric assessment of ankle and subtalar joint motion and weight-bearing radiographic assessment of talar declination and calcaneal inclination angles may aid the identification of those who have midfoot deformity that may progress to plantar midfoot ulceration.

## Competing interests

The authors declare that they have no competing interests. 

## Authors’ contributions

DRS provided concept, data collection, data analysis, writing, funding support and review for this study and manuscript. DJG provided data analysis, writing, critical review and revision for this study and manuscript. MKH provided concept support, data collection, data analysis, writing and critical review for this study and manuscript. MJS provided research design, data analysis, writing and review for this study and manuscript. KLB provided patient recruitment, patient consent, data collection, data base and retrieval and review for this study and manuscript. FWP provided technical support, data analysis software, funding support, research study design, writing and critical review for this study and manuscript. JEJ provided data collection, radiographic procedures, support for data collection, writing and critical review for this study and manuscript. All authors read and approved the final manuscript.

## Authors’ information

Mary K Hastings PT, DPT was supported by NICHD K12 HD 05593.

David J Gutekunst, PhD was supported by NICHD 5 T32 HD007434-19.

Jeffrey E Johnson MD serves as Medical Director for Musculoskeletal Applications of Shockwave Therapy for the Midwest Stone Institute (MSI). He is a stockholder in Midwest Therapy, LLC, a subsidiary company of MSI.
